# Comparison of ^18^F-FES, ^18^F-FDG, and ^18^F-FMISO PET Imaging Probes for Early Prediction and Monitoring of Response to Endocrine Therapy in a Mouse Xenograft Model of ER-Positive Breast Cancer

**DOI:** 10.1371/journal.pone.0159916

**Published:** 2016-07-28

**Authors:** SiMin He, MingWei Wang, ZhongYi Yang, JianPing Zhang, YongPing Zhang, JianMin Luo, YingJian Zhang

**Affiliations:** 1 Department of Nuclear Medicine, Fudan University Shanghai Cancer Center, Shanghai, China; 2 Department of Oncology, Shanghai Medical College, Fudan University, Shanghai, China; 3 Center for Biomedical Imaging, Fudan University, Shanghai, China; 4 Shanghai Engineering Research Center for Molecular Imaging Probes, Shanghai, China; Wayne State University, UNITED STATES

## Abstract

**Background:**

There is an increasing need to characterize biological processes for early prediction and monitoring of response to endocrine therapy in breast cancer using multiple positron emission tomography (PET) imaging probes. However, use of more than two PET tracers in a single clinical trial is quite challenging. In this study we carried out a longitudinal investigation of ^18^F-FES, ^18^F-FDG, and ^18^F-FMISO PET imaging probes for early prediction and monitoring of response to endocrine therapy in a mouse xenograft model of estrogen receptor (ER)-positive breast cancer.

**Method:**

ER^+^ human breast cancer ZR-75-1 models were established in female mice that were then randomly assigned to a treatment (fulvestrant, 5.0 mg/week for 21 days) or vehicle group. Micro-PET/CT imaging with ^18^F-FES, ^18^F-FDG, and ^18^F-FMISO was performed on days 0, 3, 14, and 21 after treatment. The uptake value (percentage injected dose per gram, %ID/g) for each probe in tumor (T) tissue and contralateral muscle (M) was measured for quantitative analysis and T/M calculation. Tumor volume was measured to record tumor growth at each time point. Tumor tissues were sampled for immunohistochemical staining of ER expression. Correlations for tumor volume and ERα levels with uptake data for the probe were tested.

**Results:**

Uptake data for ^18^F-FES in ZR-75-1 tumor tissues corresponded well with tumor response to endocrine therapy, but not for ^18^F-FDG and ^18^F-FMISO, according to longitudinal micro-PET/CT imaging and quantitative correlation analysis. There was a significant positive correlation between ^18^F-FES uptake and ER levels (%ID/g_max_ *r*^2^ = 0.76, *P*< 0.05; T/M *r*^2^ = 0.82, *P*<0.05). Notably, ^18^F-FES uptake on day 3 was significantly correlated with the day 21/baseline tumor volume ratio (%ID/g_max_ *r*^2^ = 0.74, *P* < 0.05; T/M *r*^2^ = 0.78, *P* < 0.05).

**Conclusions:**

Comparison of ^18^F-FES, ^18^F-FDG, and ^18^F-FMISO probes revealed that ^18^F-FES PET/CT molecular imaging can provide a precise early prediction of tumor response to endocrine therapy in ER^+^ breast cancer in a ZR-75-1 xenograft model. This molecular imaging strategy with ^18^F-FES PET/CT will be useful in evaluating the efficacy of endocrine therapies and in developing new endocrine drugs.

## Background

Breast cancer is one of the most common causes of cancer-related death, and approximately 75–80% of breast cancers are estrogen receptor (ER)-positive at the time of primary diagnosis [1]. Endocrine therapy has emerged as an important strategy for treating ER^+^ breast cancer. Unfortunately, approximately half of patients with breast cancer cannot benefit from endocrine therapy because of intrinsic or acquired drug resistance [2–3]. Therefore, to avoid side effects and the cost of ineffective therapy, and for more effective individualized treatment, early response prediction is crucial in determining the efficacy of various endocrine therapies for ER^+^ breast cancer.

Two methods are currently used to assess the efficacy of cancer therapy in clinical practice. The conventional method for evaluating treatment responses relies on changes in tumor size according to response evaluation criteria in solid tumors (RECIST) [4]. However, this approach has some limitations for early prediction of cancer treatment response, including a long time (many weeks to months) to reach tumor shrinkage and no reflection of changes in treatment-induced function status, especially for ER-targeted endocrine therapy. Therefore, functional molecular imaging with positron emission tomography (PET) may provide an effective way to predict response to cancer therapy since it can monitor disease-related changes in biological and chemical events using a specific molecular imaging probe in the early phase after treatment [5–7]. ^18^F-Fluorodeoxyglucose (^18^F-FDG) reflects glucose metabolism and is the most widely used PET probe for evaluating therapeutic efficiency [8]. However, ^18^F-FDG cannot be used for direct assessment of changes in ERα expression in breast cancer induced by endocrine therapy [9]. ^18^F-Fluoromisonidazole (^18^F-FMISO), an analogue of nitroimidazole, is a PET probe commonly used for hypoxic imaging as it reflects the degree of hypoxia in solid tumors in vivo [10–11]. In a previous clinical study we found that ^18^F-FMISO PET/CT could predict primary letrozole resistance in ER^+^ breast cancer [12]. However, since ^18^F-FMISO cannot target ER, this is also an indirect approach for predicting response to endocrine therapy. ^18^F-Fluoroestradiol (^18^F-FES) is a specific ER-targeted molecular probe for PET evaluation of ER expression in breast cancer [13]. Clinical studies have shown that ^18^F-FES PET can reliably detect ER^+^ breast cancer lesions and that ^18^F-FES uptake correlates well with ERα immunohistochemical (IHC) scoring [14–16]. We previously reported that ^18^F-FES PET could be used to predict the response of breast cancer to neoadjuvant chemotherapy and could help in individualizing treatment for breast cancer patients [17–18].

For early prediction and monitoring of breast cancer response to endocrine therapy, it is important to assess the use of multiple PET imaging probes. However, it is quite challenging to apply more than two PET tracers in a single clinical trial. Thus, we carried out a longitudinal molecular imaging investigation using ^18^F-FES, ^18^F-FDG, and ^18^F-FMISO PET probes for early prediction and monitoring of the response to endocrine therapy in a mouse xenograft model of ER^+^ breast cancer s in this preclinical study.

## Methods

### Ethics statement

All animal studies were conducted under a protocol approved by the Institutional Animal Care and Use Committee of Fudan University (LASFDI-20140179A032). All invasive animal procedures were performed under anesthesia (3% pentobarbital sodium 40 mg/kg) and all efforts were made to minimize suffering.

### Cell lines and mice

The ZR-75-1 human ER^+^ breast cancer cell line was purchased from Cell Bank, Shanghai Institutes for Biological Sciences, Chinese Academy of Sciences. The cells were grown in RPMI 1640 medium with L-glutamine, penicillin 100 μg/mL, streptomycin 100 μg/mL and 10% fetal calf serum in a humidified 5% CO_2_ atmosphere at 37°C. Female BALB/c nude mice aged 4–6 weeks were purchased from the Department of Laboratory Animal Science, Fudan University, and housed in laminar flow cabinets under specific pathogen-free conditions and provided with food and water ad libitum. During the entire study period, the body weight and behaviors of mice were monitored by a balance and visual observation every two day. No significant body loss and abnormal behaviors were observed. The mice were sacrificed by overdose of anesthesia at each imaging time point, at the study end, or when the maximum tumor size in long diameter reached 20 mm.

### Xenograft model of human breast cancer

Estrogen pellets (0.72 mg, 90-day release, Innovative Research, USA) were implanted into the body of mice 3 days before tumor cell inoculation and were left until the tumors reached 6.0–8.0 mm (~14 days after inoculation). ZR-75-1 (5×10^6^ cells in 100 μL of medium mixed with 100 μL of Matrigel (BD Biosciences)) were injected into the mammary fat pad on the right thorax of mice. All invasive procedures were performed under anesthesia (3% pentobarbital sodium 40 mg/kg). Tumor growth was followed via caliper measurement of the perpendicular axes. Tumor volume was calculated as *V* = α × (*b*^2^)/2 [19], where *α* is tumor length and *b* is tumor width.

### Endocrine therapy

When tumors had grown to 6–8 mm in diameter, mice were randomly assigned to a treatment or vehicle group (*n* = 10 per group), and the estrogen pellets were surgically removed before treatment initiation. The dose and protocol for each group were as follows: vehicle, 0.9% sodium chloride, 50 μl/mouse/week, s.c.; and fulvestrant, 5 mg/mouse/weekly, s.c., prepared according to standard methods. The treatment duration was 21 days.

### Micro-PET/CT imaging and quantitative analysis

The ^18^F-FES, ^18^F-FDG, and ^18^F-FMISO PET probes were produced using a modified Explora FDG_4_ module (Siemens) in our center as previously reported [20–21]. Micro-PET/CT (Inveon, Siemens) scanning was performed on days 0, 3, 14, and 21 after treatment with injection of 5.55 MBq (150 μCi) of ^18^F-FES, ^18^F-FDG, or ^18^F-FMISO into the tail vein. The animal numbers for each PET probe were 10, 9, 8, and 7 on days 0, 3, 14, and 21, respectively, because of tumor sampling for IHC. Considering ^18^F decay, microPET/CT imaging were performed in the order of ^18^F-FDG, ^18^F-FES and ^18^F-FMISO with 16-20h interval for a specific imaging time point [22]. Before ^18^F-FDG administration, mice were kept fasting for at least 6.0 h. ^18^F-FMISO was injected 90 min before the scan start [23], and ^18^F-FES and ^18^F-FDG 60 min before the scan start. Isoflurane was administered 10 minutes before the scan start, and mice were maintained under anesthesia during the scanning period. The images were reconstructed using a three-dimensional ordered-subset expectation maximization (OSEM3D)/maximum algorithm. For data analysis, the region of interest (ROI) was manually drawn to cover the whole tumor on fused images. A similar circular ROI was drawn on the muscle of the opposite foreleg of the mouse on fused images. The percentage injected dose per gram (%ID/g) in the tumor and muscle ROIs was recorded. The tumor-to-muscle ratio (T/M) was calculated by dividing %ID/g for tumor tissue by that for muscle. T/M before and after therapy were denoted as T/M day_0_ and T/M day_*n*_, and %ID/g as %ID/g day_0_ and %ID/g day_*n*_, respectively. Changes after therapy are denoted as ΔT/M = (T/M day_*n*_ − T/M day_0_)/ T/M day_0_ × 100% and Δ%ID/g = (%ID/g day_*n*_ − %ID/g day_0_)/ %ID/g day_0_ × 100%. [10].

### Immunohistochemistry

IHC for ERα was performed using standard protocols according to the manufacturers’ instructions (Santa Cruz Biotechnology, TX, USA), where the tumor tissues were sampled from both treatment and control groups at each imaging time point. IHC analysis for quantitative ERα expression was based on standard procedures for breast cancer [24]. The total proportion of cells positively stained any intensity was scored as follows: 0, no cells stained; 1, 1%–25% cells stained; 2, 26%–50% cells stained; 3, 50%–75% cells stained; and 4, >75% cells stained.

### Statistical analysis

Data are expressed as mean ± SD. Within-group comparisons (before and after treatment) and differences among ER^+^ breast cancer groups were assessed using analysis of variance (ANOVA) models. An unpaired *t*-test was used to determine statistical significance between experimental and control groups. Pearson correlation coefficients were calculated to determine the association between treatment-induced changes in^18^F-FES uptake and ER expression according to IHC. *P* values < 0.05 were considered statistically significant. All statistical analyses were performed using SPSS version 19.0 (SPSS-IBM).

## Results

### Impact of endocrine therapy on ER^+^ breast cancer growth

**Fig 1:**
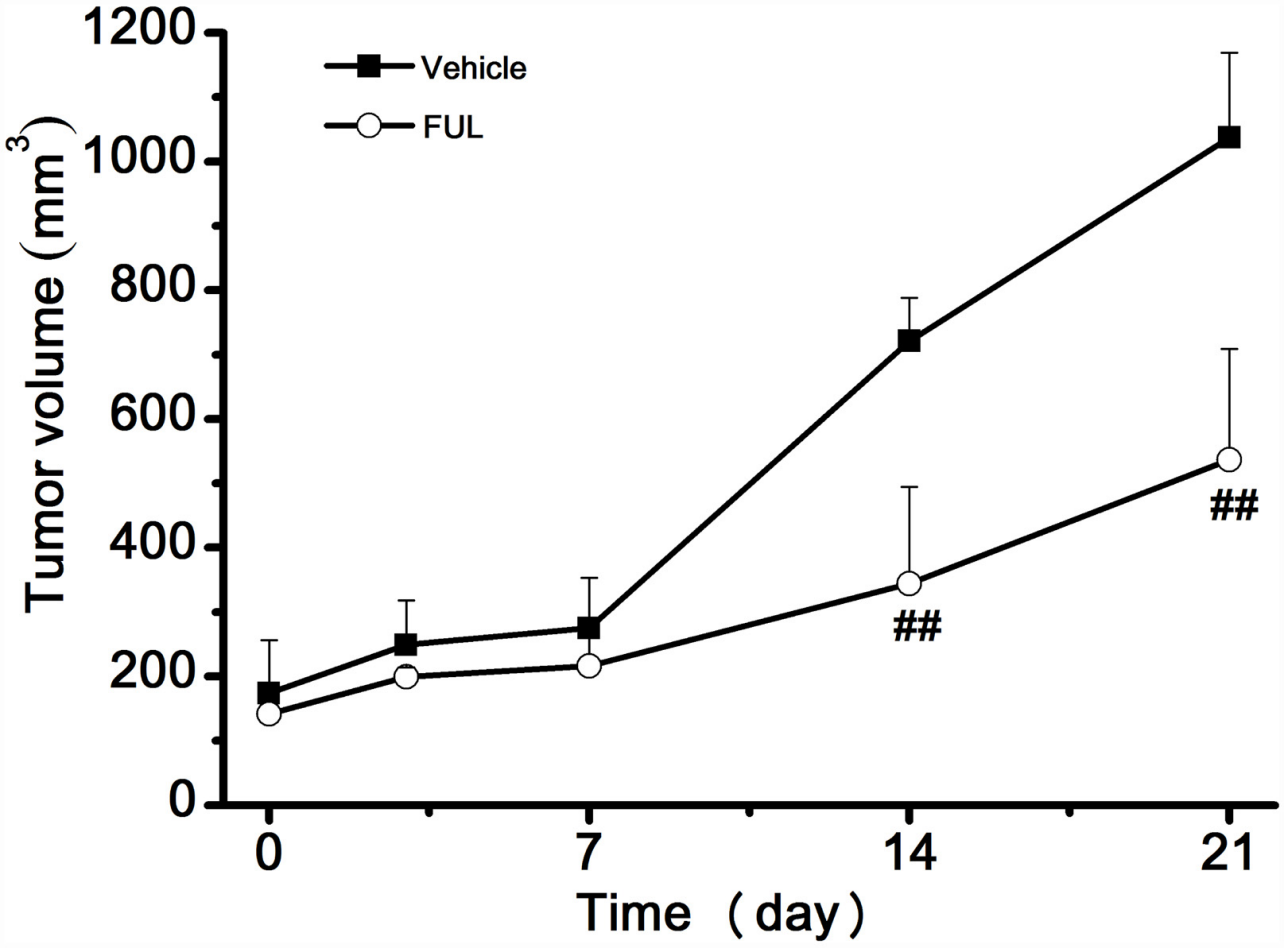
Effect of endocrine therapy on the growth of ZR-75-1 xenografts. ** *P*< 0.001 compared to the vehicle group.

The changes in tumor volume are shown in Fig 1. There was no significant difference in tumor volume between the treatment and vehicle groups from day 0 to day 7 (*P* > 0.05), but a significant difference was observed between the groups on days 14 and 21 (*P* < 0.001).

### ^18^F-FES micro-PET/CT imaging during endocrine therapy for ER^+^ breast cancer

**Fig 2:**
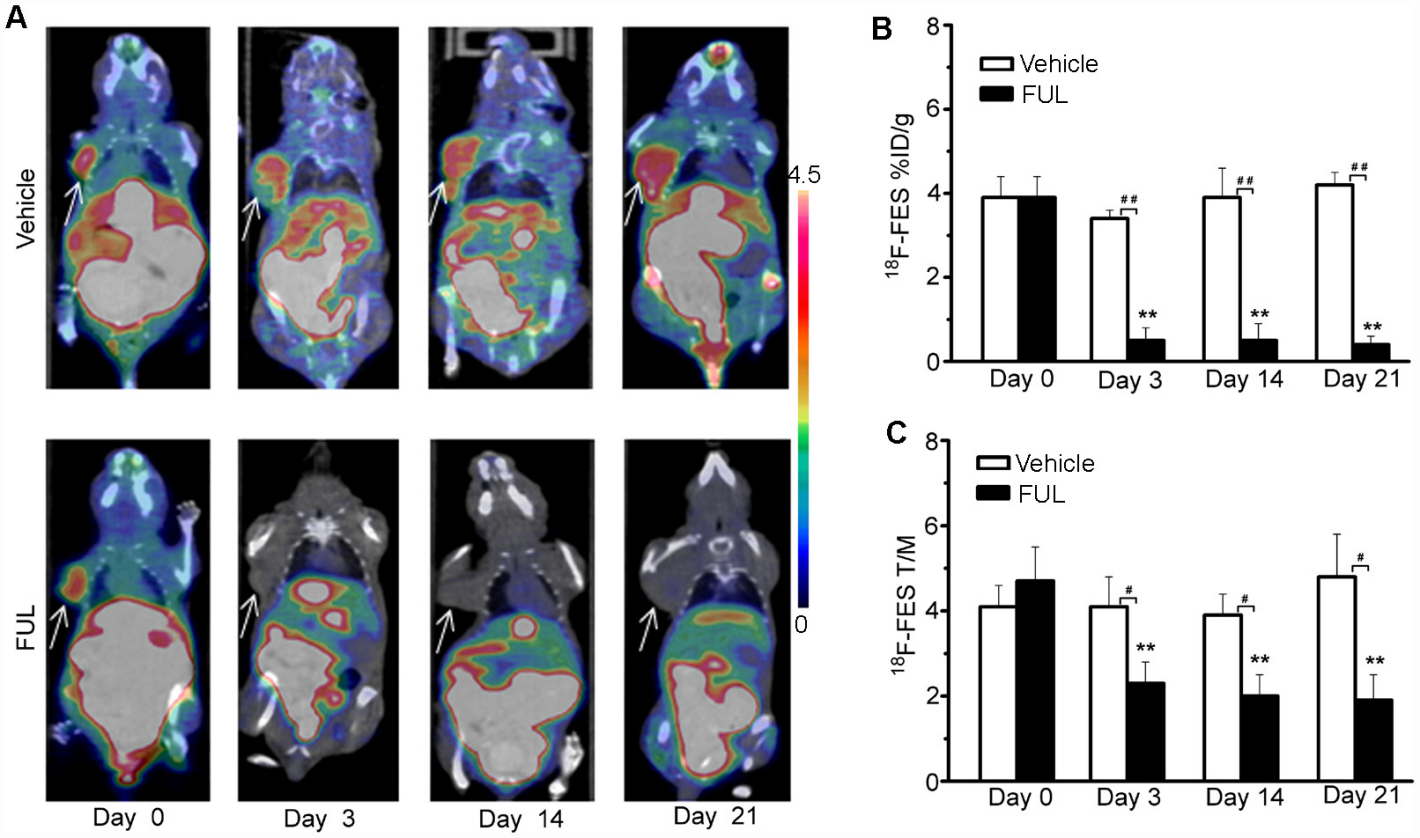
^18^F-FES PET/CT imaging of ZR-75-1 tumor-bearing mice. (A) Representative ZR-75-1 tumors were imaged using PET/CT with ^18^F-FES on days 0, 3, 14, and 21 after treatment. The tumors are indicated by arrows. (B, C) ^18^F-FES PET/CT analysis (%ID/g_max_ and T/M) on days 0, 3, 14, and 21 after treatment. ** *P*< 0.001, within groups compared to baseline; # *P*< 0.05, ## *P*< 0.001 between treatment and vehicle groups.

The uptake of ^18^F-FES by ZR-75-1 tumor tissues corresponded well to the efficacy of endocrine therapy according to longitudinal ^18^F-FES micro-PET/CT imaging (Fig 2A). As shown in Fig 2B, ^18^F-FES %ID/g_max_ in the vehicle group showed no significant change compared with baseline at any time point (*P* > 0.05). By contrast, tumor uptake of ^18^F-FES in the treatment group decreased remarkably from day 3. ^18^F-FES %ID/g_max_ significantly decreased on day 3 (−86 ± 8%, *P* < 0. 001), day 14 (− 86 ± 8%, *P* <0.001), and day 21 (−87 ± 5%, *P* < 0.001) compared to baseline. Moreover, significant differences in ^18^F-FES tumor uptake between the groups were observed on days 3, 14, and 21 (*P* < 0.001). T/M ratios for ^18^F-FES exhibited similar results to those for %ID/g_max_ (Fig 2C).

### ^18^F-FDG micro-PET/CT imaging during endocrine therapy for ER^+^ breast cancer

**Fig 3:**
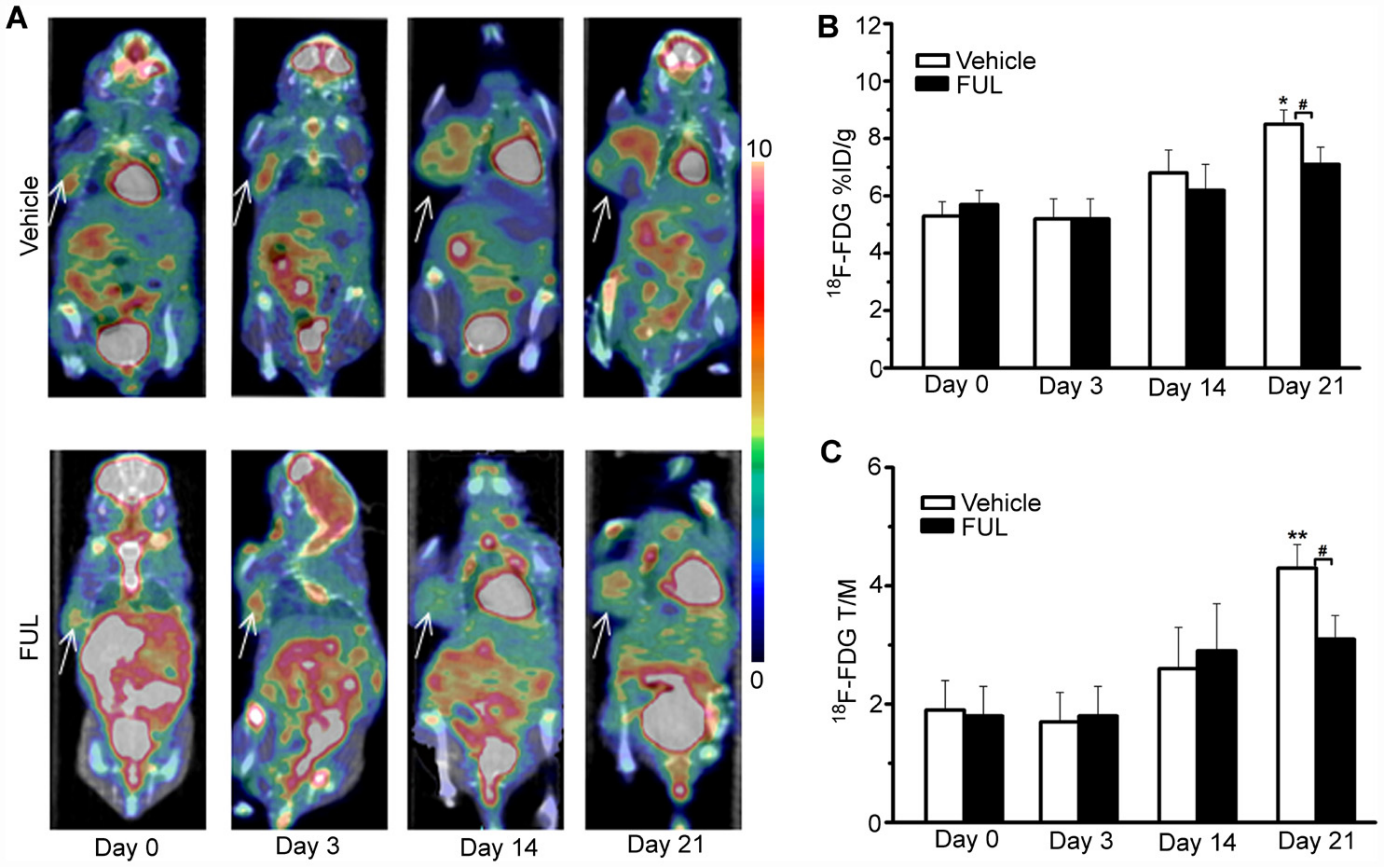
^18^F-FDG PET/CT imaging of ZR-75-1 tumor-bearing mice. (A) Representative ZR-75-1 tumors were imaged using PET/CT with ^18^F-FDG on days 0, 3, 14, and 21 after treatment. The tumors are indicated by arrows. (B, C) ^18^F-FDG PET/CT analysis (%ID/g_max_ and T/M) on days 0, 3, 14, and 21 after treatment. * *P*< 0.05, ** *P*< 0.001, within groups compared to baseline; # *P*< 0.05 between treatment and vehicle groups.

Longitudinal ^18^F-FDG micro-PET/CT imaging (Fig 3A) was performed during endocrine therapy for ER^+^ breast cancer in ZR-75-1 xenografts since ^18^F-FDG is routinely used to follow cancer response to therapy. We found no clear relationship between ^18^F-FDG uptake by ZR-75-1 tumor tissues and endocrine therapy efficacy according to longitudinal ^18^F-FDG Micro-PET/CT imaging (Fig 3). ^18^F-FDG %ID/g_max_ values in the vehicle group showed a slight increase from day 0 to day 3 (+6 ± 10%, *P* > 0.05), day 14 (+47 ± 21%, *P* > 0.05), and day 21 (+67 ± 20%, *P* < 0. 05) compared to baseline (Fig 3B). In the treatment group, ^18^F-FDG %ID/g_max_ fluctuated within a very narrow range and there was no significant difference compared to baseline at any time point (*P* > 0. 05) (Fig 3B). Comparisons between the vehicle and treatment groups revealed no significant difference at the early time points, but a significant difference in ^18^F-FDG uptake was observed between the groups on day 21. T/M ratios for ^18^F-FDG within and between the groups showed similar results to those for %ID/g_max_ (Fig 3C).

### ^18^F-FMISO micro-PET/CT imaging during endocrine therapy for ER^+^ breast cancer

**Fig 4:**
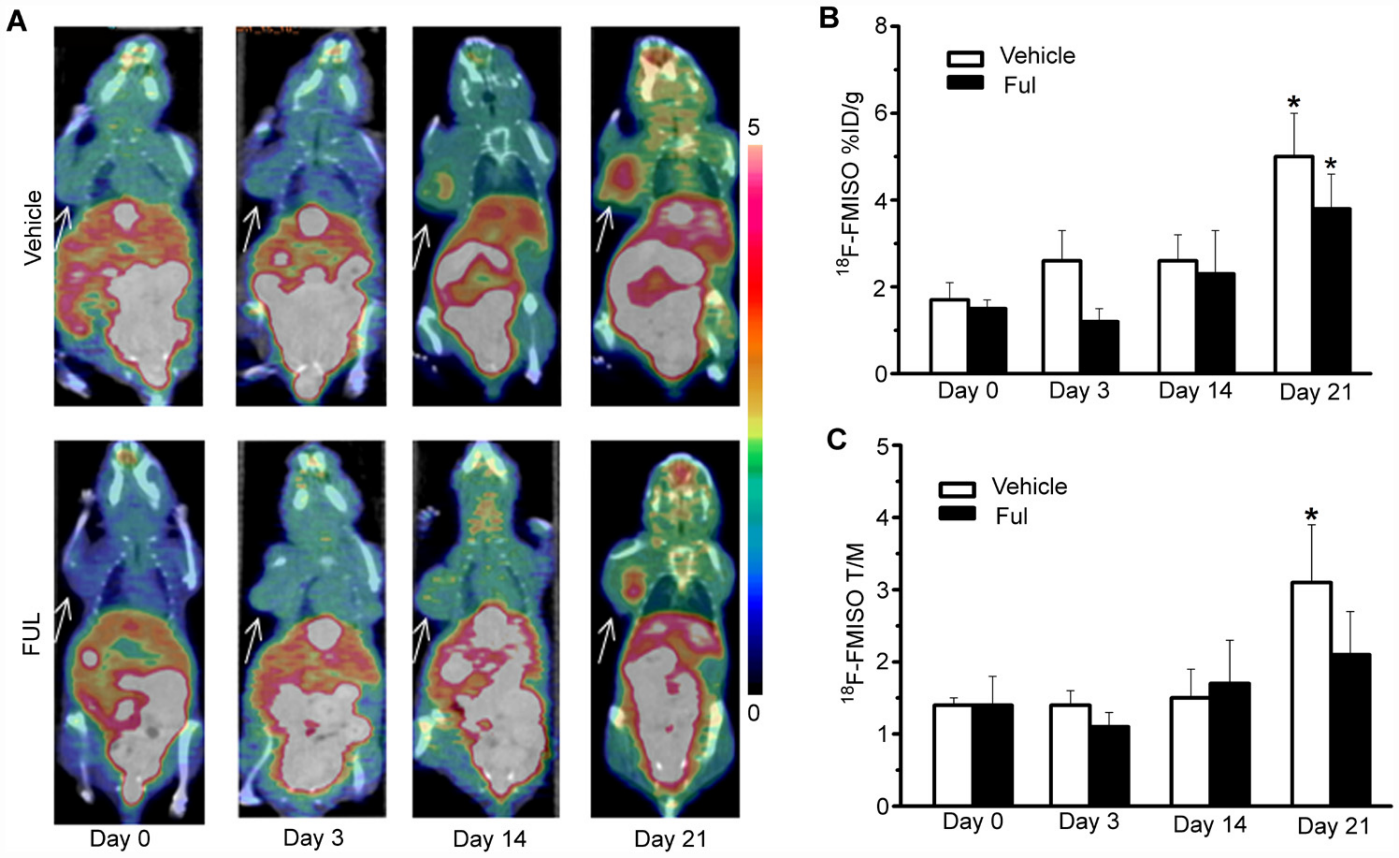
^18^F-FMISO PET/CT imaging of ZR-75-1 tumor-bearing mice. (A) Representative ZR-75-1 tumors were imaged using PET/CT with ^18^F-FMISO on days 0, 3, 14, and 21 after treatment. The tumors are indicated by arrows. (B, C) ^18^F-FMISO PET/CT analysis (%ID/g_max_ and T/M) on days 0, 3, 14, and 21 after treatment. * *P*< 0.05, within groups compared to baseline.

Longitudinal ^18^F-FMISO micro-PET/CT imaging (Fig 4A) was investigated during endocrine therapy for ER^+^ breast cancer in ZR-75-1 xenografts because of its suitability for determining hypoxia in solid tumors. Longitudinal ^18^F-FMISO micro-PET/CT imaging (Fig 4) revealed no significant relationship between ^18^F-FMISO uptake and endocrine therapy response. As shown in Fig 4B, ^18^F-FMISO %ID/g_max_ values for the vehicle group showed a slight increase from day 0 to day 3 (+46 ± 10%, *P* > 0.05), day 14 (+59 ± 21%, *P* > 0.05), and day 21(+219 ± 20%, *P* < 0. 05) compared to baseline. In the treatment group, a similar trend was observed from day 0 to day 3 (−17 ± 10%, *P* > 0.05), day 14 (+61 ± 21%, *P* > 0.05), and day 21 (+153 ± 20%, *P* < 0. 05). Comparison of the vehicle and therapy groups revealed no significant difference in ^18^F-FMISO uptake at any time point after endocrine therapy (*P* > 0.05). For^18^F-FMISO T/M ratios within and between the groups, similar results to those for %ID/g_max_ were observed (Fig 4C).

### Correlation of ^18^F-FES %ID/g_max_ with ERα level in tumor tissues

**Fig 5:**
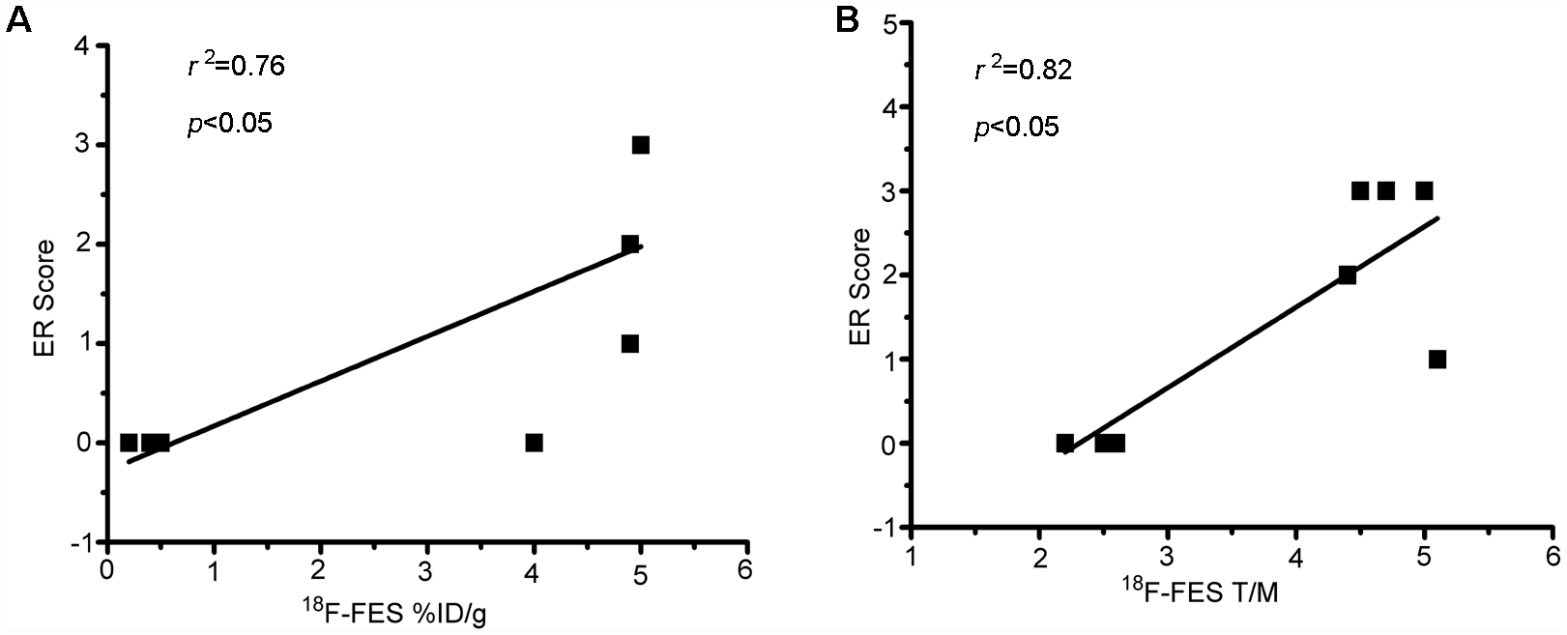
^18^F-FES uptake in comparison to IHC results. There is a significant positive correlation between ^18^F-FES uptake and ER expression (%ID/g_max_, *r*^2^ = 0.76, *P* < 0.05; T/M, *r*^2^ = 0.82, *P* < 0.05).

ERα IHC was scored for quantitative determination of the expression level of ERα in ZR-75-1 tumor tissues at each designated time point. Fig 5 shows that there was a significant positive correlation between ^18^F-FES uptake and ERα expression in terms of %ID/g_max_ (*r*^2^ = 0.76, *P* < 0.05, Fig 5A) and T/M (*r*^2^ = 0.82, *P* < 0.05, Fig 5B).

### Correlation between PET and tumor growth

**Fig 6:**
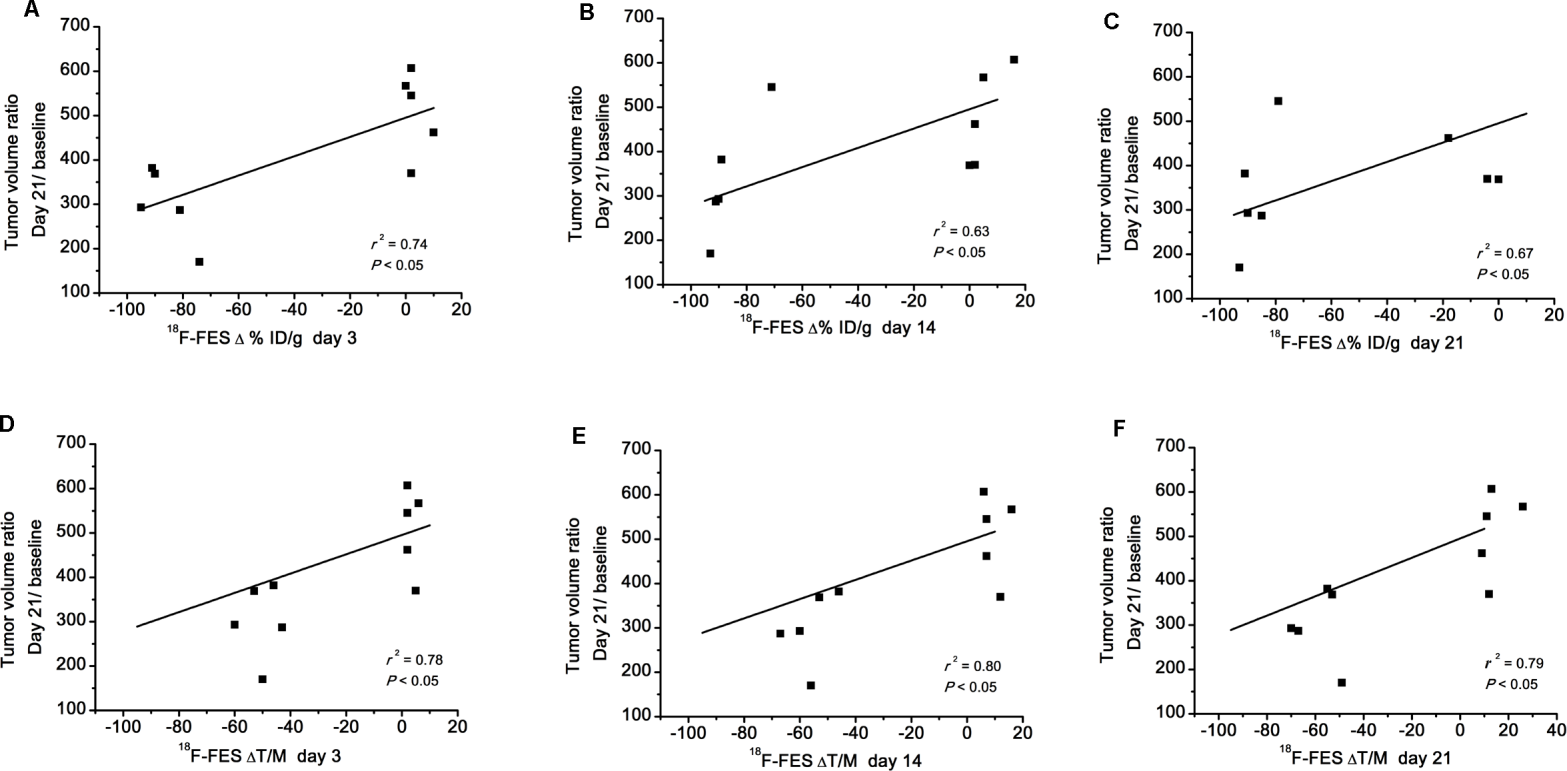
Correlation between tumor ^18^F-FES uptake and tumor growth. ^18^F-FES Δ%ID/g_max_ and ΔT/M on day 3, day 14 and day 21 was significantly correlated with the day 21/baseline tumor volume ratio.

To determine whether ^18^F-FES uptake might predict tumor response, we investigated the relationship between probe uptake and changes in tumor volume (Fig 6). Both ^18^F-FES Δ%ID/g (*r*^2^ = 0.74, *P* < 0.05, Fig 6A; *r*^2^ = 0.63, *P* < 0.05, Fig 6B; *r*^2^ = 0.67, *P* < 0.05, Fig 6C) and ΔT/M (*r*^2^ = 0.78, *P*< 0.05, Fig 6D; *r*^2^ = 0.80, *P* < 0.05, Fig 6E; *r*^2^ = 0.79, *P* < 0.05, Fig 6F) on day 3, day 14 and day 21 were significantly correlated with the day 21/baseline tumor volume ratio.

## Discussion

Fulvestrant, a selective ER downregulator, is approved for treatment of locally advanced or metastatic ER^+^ breast cancer [25]. However, clinical dilemmas remain because approximately half of breast cancer patients do not benefit from fulvestrant because of intrinsic or acquired resistance. Therefore, early and precise prediction of tumor responsiveness to endocrine therapy is highly valuable for identifying ER^+^ breast cancer patients who will require a change in treatment strategy.

We demonstrated the feasibility of ^18^F-FES PET/CT use for early and precise prediction of the efficacy of fulvestrant therapy in a model of ER^+^ breast cancer. The treatment-induced inhibition of tumor growth was in accordance with early changes observed on ^18^F-FES PET. Fulvestrant significantly inhibited tumor growth in ZR-75-1 breast cancer (Fig 1). As a result, tumor ^18^F-FES uptake in the fulvestrant group decreased remarkably compared to the vehicle group on day 3. The significant decrease in ^18^F-FES uptake could be largely explained by reduced ER expression due to protein ubiquitination and degradation and/or direct blockade of ER by fulvestrant [26]. By contrast, ^18^F-FES uptake in the vehicle group showed no significant change from baseline at any time point. The PET findings for ^18^F-FES uptake were confirmed by ER IHC. We further analyzed the relationship between changes in ^18^F-FES uptake and tumor volume. Δ%ID/g and ΔT/M for ^18^F-FES on day 3 were significantly correlated with the change in tumor growth on day 21 compared to baseline (*r*^2^ = 0.78, *P*< 0.001; *r*^2^ = 0.61, *P*< 0.001). This result indicates that quantitative changes in ^18^F-FES uptake by tumor tissue could be an early predictor of responders to endocrine therapy in ER^+^ breast cancer. Most importantly, changes in ^18^F-FES uptake occurred before measurable changes in tumor size, which could be beneficial for evaluation of endocrine therapies for ER^+^ breast cancer patients in translational investigations in the near future.

Apart from the between-group findings, longitudinal ^18^F-FES PET imaging can measure the pharmacodynamics of endocrine drugs in ER^+^ breast cancer over time. Tumors treated with fulvestrant showed the most significant decrease in ^18^F-FES %ID/g_max_ and T/M on day 3 after treatment. However, tumor ^18^F-FES uptake days 14 and 21 did not differ from that on day 3 (*P* > 0.05). By contrast, tumor ^18^F-FES uptake in the vehicle group gradually increased during the course of treatment. Therefore, ^18^F-FES PET/CT imaging offers a noninvasive way to determine quantitatively changes in the tumor oestrogen receptor during endocrine therapy.

Currently, ^18^F-FES PET/CT has brought clinical benefits to breast cancer patients by making appropriate hormonal treatment based on the measurement of oestrogen receptor [27–28]. However, factors those affect tumor ^18^F-FES uptake appear complex and multiple aspects, such as concomitant therapies and menopausal status [29]. For example, Fowler et al. reported that ^18^F-FES uptake decreased in fulvestrant-resistant tumor model after endocrine therapy [30]. To address this medical issue, more efforts should be payed from basic researches and clinical trials, such as those in this study and the ongoing trial [31].

Finally, our findings indicate that ^18^F-FES was superior to ^18^F-FDG and ^18^F-FMISO as a PET imaging probe in predicting response to endocrine therapy in ER^+^ breast cancer. According to Fig 3, there was no significant difference in ^18^F-FDG uptake by ER^+^ breast cancer between the treatment and vehicle groups until day 21, so ^18^F-FDG PET could not differentiate between responders and non-responders to endocrine therapy in the early phase. The reason might be that glucose metabolism is not directly affected by endocrine therapy in breast cancer and thus a longer time might be required for significant outcomes [9]. Similarly, quantitative values (%ID/g_max_, T/M) for ^18^F-FMISO uptake in the treatment group generally exhibited a transient decrease on day 3 and a continuous increase thereafter. However, there was no significant difference in ^18^F-FMISO uptake by ER^+^ breast cancer between the vehicle and therapy groups. Thus, ^18^F-FDG and ^18^F-FMISO as PET probes are not the first choice for prediction of early response to endocrine therapy for ER^+^ breast cancer.

Our study has several limitations. First, IHC of Glut-1 and HIF-1 in relation to ^18^F-FDG and ^18^F-FMISO was not performed because there was no correlation between their uptake and treatment response. Second, only a single cell line and one model of breast cancer were used. Third, the role of ^18^F-FDG and ^18^F-FMISO PET/CT in evaluating endocrine therapy response of breast cancer was not as good as that we expected, which indicated that more research efforts should be paid on multiple PET imaging probe strategy for diagnosis and therapy of breast cancer. Further studies are needed to investigate clinical translation of PET molecular imaging for response prediction and follow-up of endocrine therapies for different breast cancer mechanisms.

## Conclusions

Comparison of ^18^F-FES, ^18^F-FDG, and ^18^F-FMISO probes showed that ^18^F-FES PET/CT imaging is suitable for precise and early prediction and monitoring of response to endocrine therapy in ER^+^ breast cancer. Among the three probes evaluated, ^18^F-FES will provide the greatest benefit in evaluating the efficacy evaluation of endocrine therapy.

## Supporting Information

S1 File Data of tumor volume (mm^3^). Table A and Table B in S1 File are the tumor volume changes in vehicle and fulvestrant groups at different times, respectively.
(PDF)

S2 File ^18^F-FES MicroPET/CT imaging and quantitative value (%ID/g_max_, T/M). Figure A and Figure B in S2 File are ^18^F-FES PET/CT images of vehicle and fulvestrant groups on days 0, 3, 14, and 21 after treatment, respectively. Table A and Table B in S2 File are the value of ^18^F-FES %ID/g_max_ in vehicle and fulvestrant groups, respectively. Table C and Table D in S2 File are the value of ^18^F-FES T/M in vehicle and fulvestrant groups, respectively.
(PDF)

S3 File ^18^F-FDG MicroPET/CT imaging and quantitative value (%ID/g_max_, T/M). Figure A and Figure B in S3 File are ^18^F-FDG PET/CT images of vehicle and fulvestrant groups on days 0, 3, 14, and 21 after treatment, respectively. Table A and Table B in S3 File are the value of ^18^F-FDG %ID/g_max_ in vehicle and fulvestrant groups, respectively. Table C and Table D in S3 File are the value of ^18^F-FDG T/M in vehicle and fulvestrant groups, respectively.
(PDF)

S4 File ^18^F-FMISO MicroPET/CT imaging and quantitative value (%ID/g_max_, T/M). Figure A and Figure B in S4 File are ^18^F-FMISO PET/CT images of vehicle and fulvestrant groups on days 0, 3, 14, and 21 after treatment, respectively. Table A and Table B in S4 File are the value of ^18^F-FMISO %ID/g_max_ in vehicle and fulvestrant groups, respectively. Table C and Table D in S4 File are the value of ^18^F-FMISO T/M in vehicle and fulvestrant groups, respectively.
(PDF)

S1 Table Correlation data of ER score and ^18^F-FES uptake value (%ID/g_max_, T/M). (PDF)
