# Supplementary material for: Comparison of 18F-FES, 18F-FDG, and 18F-FMISO PET Imaging Probes for Early Prediction and Monitoring of Response to Endocrine Therapy in a Mouse Xenograft Model of ER-Positive Breast Cancer
Source: PLoS One. 2016 Jul 28;11(7):e0159916. doi: 10.1371/journal.pone.0159916 (PMC4965120; doi:10.1371/journal.pone.0159916)
Supplement: S1 File — Table A and Table B in S1 File are the tumor volume changes in vehicle and fulvestrant groups at different times, respectively. (PDF) [file pone.0159916.s001.pdf]

S1 File. Data of tumor volume (mm^3).

Table A. Data of tumor volume in vehicle group

Vehicle Group	Mice 1	Mice 2	Mice 3	Mice 4	Mice 5	Mice 6
d0	184	212	190	287	40	135
d3	267	313	183	346	186	203
d7	322	214	213	310	394	200
d14	786	714	746	700	780	602
d21	1228	857	1069	1130	988	955

Table B. Data of tumor volume in fulvestrant group

Fulvestrant Group	Mice 1	Mice 2	Mice 3	Mice 4	Mice 5	Mice 6
d0	145	130	120	203	100	157
d3	186	188	224	206	207	186
d7	245	225	214	216	269	127
d14	303	303	626	328	336	171
d21	562	610	774	550	482	247
