# Supplementary material for: Comparison of 18F-FES, 18F-FDG, and 18F-FMISO PET Imaging Probes for Early Prediction and Monitoring of Response to Endocrine Therapy in a Mouse Xenograft Model of ER-Positive Breast Cancer
Source: PLoS One. 2016 Jul 28;11(7):e0159916. doi: 10.1371/journal.pone.0159916 (PMC4965120; doi:10.1371/journal.pone.0159916)
Supplement: S2 File — Figure A and Figure B in S2 File are 18F-FES PET/CT images of vehicle and fulvestrant groups on days 0, 3, 14, and 21 after treatment, respectively. Table A and Table B in S2 File are the value of 18F-FES %ID/gmax in vehicle and fulvestrant groups, respectively. Table C and Table D in S2 File are the value of 18F-FES T/M in vehicle and fulvestrant groups, respectively. (PDF) [file pone.0159916.s002.pdf]

S2 File. ^{18}F-FES MicroPET/CT imaging and quantitative value (%ID/g_{max}, T/M).

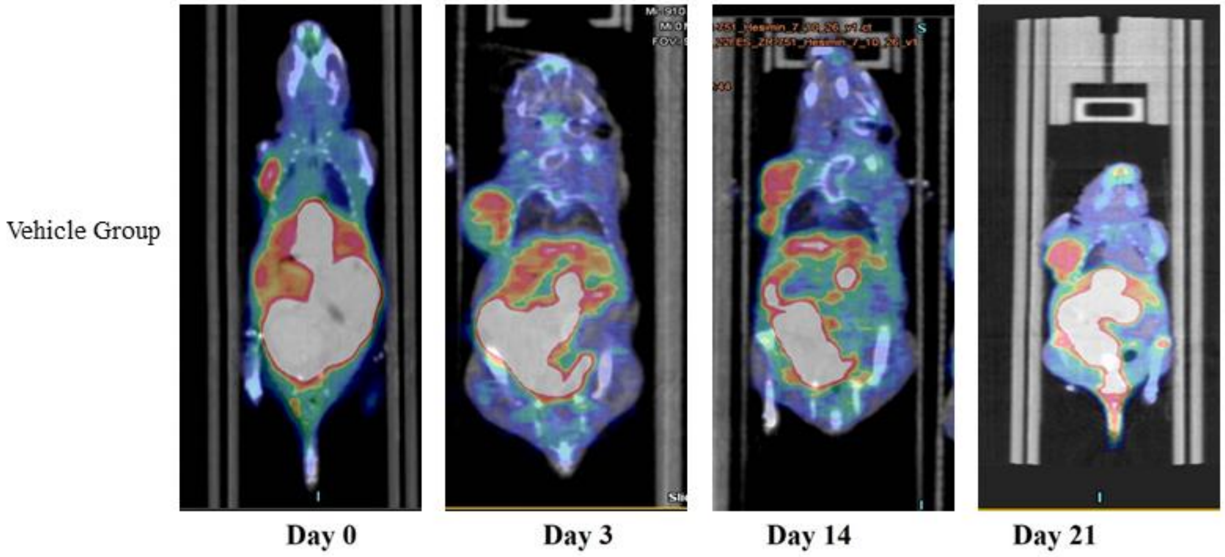

Figure A.[18]F-FES MicroPET/CT imaging in vehicle group

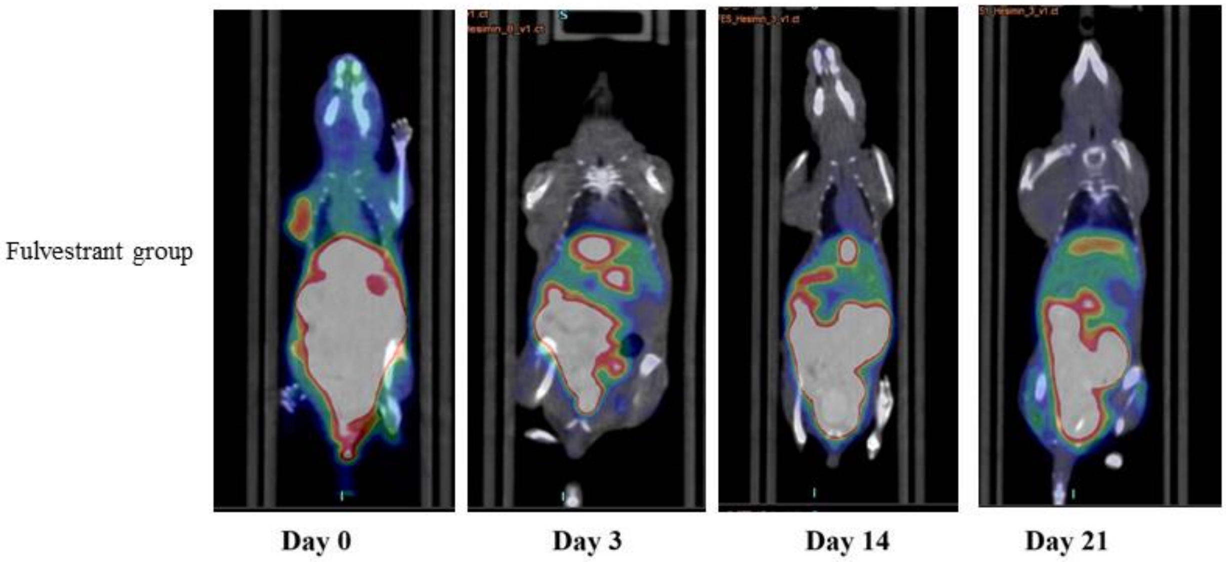

Figure B.[18]F-FES MicroPET/CT imaging in fulvestrant group

Table A. Quantitative value of ^{18}F-FES %ID/g_{max} in vehicle group

Vehicle Group	%ID/g_{max} of ^{18}F-FES			
	Day 0	Day 3	Day 14	Day 21
Mice 1	3.9	3.9	3.9	3.9
Mice 2	3	3.3	3.5	4.8
Mice 3	3.4	3.5	3.6	4.2
Mice 4	4.4	4.5	4.5	4.2
Mice 5	4.9	5	5	4

Table B. Quantitative value of ^{18}F-FES %ID/g_{max} in fulvestrant group

Fulvestrant Group	%ID/g_{max} of ^{18}F-FES			
	Day 0	Day 3	Day 14	Day 21
Mice 1	3.9	1	1.1	0.8
Mice 2	3	0.3	0.3	0.3
Mice 3	3.4	0.3	0.3	0.5
Mice 4	4.4	0.8	0.3	0.3
Mice 5	4.9	0.2	0.5	0.4

Table C. Quantitative value of ^{18}F-FES T/M in vehicle group

Vehicle Group	T/M of ^{18}F-FES			
	Day 0	Day 3	Day 14	Day 21
Mice 1	3	3.2	3.5	3.8
Mice 2	4.2	4.3	4.5	4.6
Mice 3	4.2	4.3	4.5	4.7
Mice 4	4	4.2	4.5	4.5
Mice 5	4.4	4.5	4.7	5

Table D. Quantitative value of ^{18}F-FES T/M in fulvestrant group

**Fulvestrant Group**	**T/M of ^{18}F-FES**			
	Day 0	Day 3	Day 14	Day 21
Mice 1	5.1	2.5	2.2	2.6
Mice 2	3.2	1.5	1.5	1.5
Mice 3	5.6	3	3	2.5
Mice 4	4.6	2.6	1.5	1.5
Mice 5	5	2	2	1.5
