# Supplementary material for: Comparison of 18F-FES, 18F-FDG, and 18F-FMISO PET Imaging Probes for Early Prediction and Monitoring of Response to Endocrine Therapy in a Mouse Xenograft Model of ER-Positive Breast Cancer
Source: PLoS One. 2016 Jul 28;11(7):e0159916. doi: 10.1371/journal.pone.0159916 (PMC4965120; doi:10.1371/journal.pone.0159916)
Supplement: S3 File — Figure A and Figure B in S3 File are 18F-FDG PET/CT images of vehicle and fulvestrant groups on days 0, 3, 14, and 21 after treatment, respectively. Table A and Table B in S3 File are the value of 18F-FDG %ID/gmax in vehicle and fulvestrant groups, respectively. Table C and Table D in S3 File are the value of 18F-FDG T/M in vehicle and fulvestrant groups, respectively. (PDF) [file pone.0159916.s003.pdf]

S3 File. ^{18}F-FDG MicroPET/CT imaging and quantitative value (%ID/g_{max}, T/M).

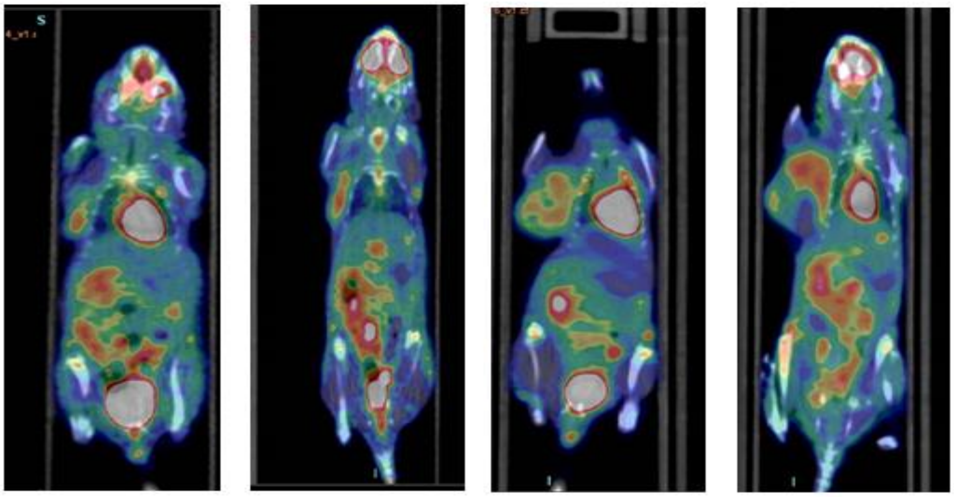

Figure A. ^{18}F-FDG MicroPET/CT imaging in vehicle group

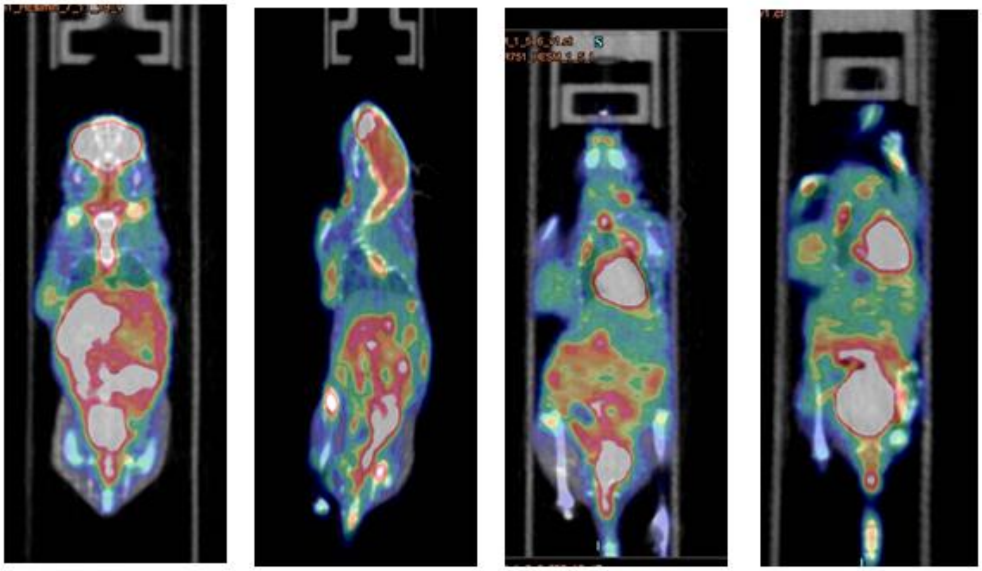

Figure B. ^{18}F-FDG MicroPET/CT imaging in fulvestrant group

Table A. Quantitative value of ^{18}F-FDG %ID/g_{max} in vehicle group

Vehicle Group	%ID/g_{max} of ^{18}F-FDG			
	Day 0	Day 3	Day 14	Day 21
Mice 1	6.1	7.1	7.2	8.2
Mice 2	5	3.1	7.7	8.2
Mice 3	4.2	5.2	6.1	8.2
Mice 4	6.8	7.3	6.9	8.9
Mice 5	4.4	3.4	6.3	9

Table B. Quantitative value of ^{18}F-FDG %ID/g_{max} in fulvestrant group

Fulvestrant Group	%ID/g_{max} of ^{18}F-FDG			
	Day 0	Day 3	Day 14	Day 21
Mice 1	6	7.1	6.8	7.9
Mice 2	4.4	3.1	5	6.2
Mice 3	4.2	5.2	6	7.6
Mice 4	6.9	6.3	8.3	6.2
Mice 5	7.2	4.4	5	7.7

Table C. Quantitative value of ^{18}F-FDG T/M in vehicle group

Vehicle Group	T/M of ^{18}F-FDG			
	Day 0	Day 3	Day 14	Day 21
Mice 1	1.8	2.6	2.3	5.1
Mice 2	1.4	1.2	1.4	3.9
Mice 3	2.3	1.6	3.1	4.8
Mice 4	2.7	1.3	3.8	3.1
Mice 5	1.4	1.9	2.6	4.9

Table D. Quantitative value of ^{18}F-FDG T/M in fulvestrant group

**Vehicle Group**	**T/M of ^{18}F-FDG**			
	Day 0	Day 3	Day 14	Day 21
Mice 1	2.8	2.6	3.2	3.4
Mice 2	1	1.2	1.2	2.9
Mice 3	1.1	1.6	3.7	2.6
Mice 4	2	1.3	5.5	3.8
Mice 5	2.4	2.3	1.2	3.1
