# Supplementary material for: Comparison of 18F-FES, 18F-FDG, and 18F-FMISO PET Imaging Probes for Early Prediction and Monitoring of Response to Endocrine Therapy in a Mouse Xenograft Model of ER-Positive Breast Cancer
Source: PLoS One. 2016 Jul 28;11(7):e0159916. doi: 10.1371/journal.pone.0159916 (PMC4965120; doi:10.1371/journal.pone.0159916)
Supplement: S4 File — Figure A and Figure B in S4 File are 18F-FMISO PET/CT images of vehicle and fulvestrant groups on days 0, 3, 14, and 21 after treatment, respectively. Table A and Table B in S4 File are the value of 18F-FMISO %ID/gmax in vehicle and fulvestrant groups, respectively. Table C and Table D in S4 File are the value of 18F-FMISO T/M in vehicle and fulvestrant groups, respectively. (PDF) [file pone.0159916.s004.pdf]

S4 File. ^{18}F-FMISO MicroPET/CT imaging and quantitative value (%ID/g_{max}, T/M).

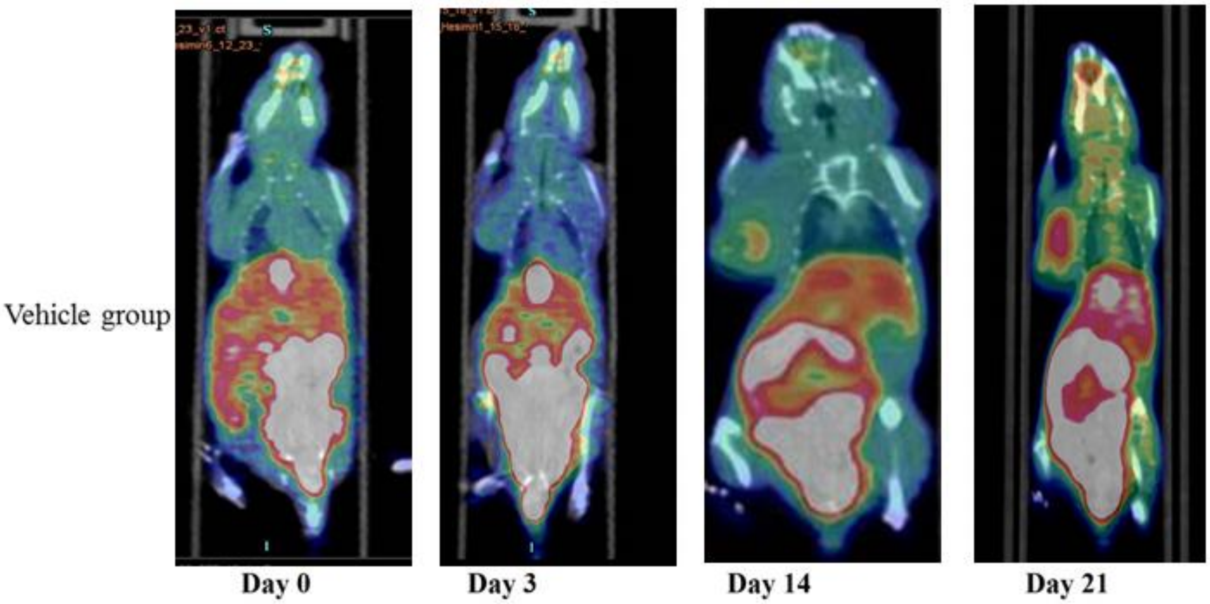

Figure A.^{18}F-FMISO MicroPET/CT imaging in vehicle group

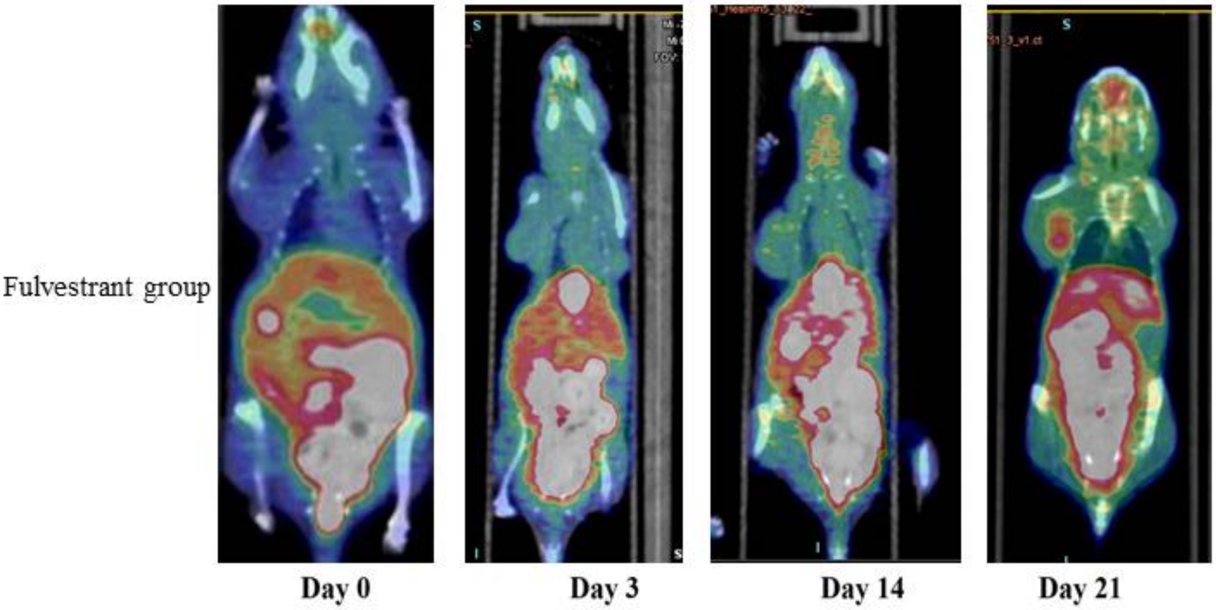

Figure B.^{18}F-FMISO MicroPET/CT imaging in fulvestrant group

Table A. Quantitative value of ^{18}F-FMISO %ID/g_{max} in vehicle group

Vehicle Group	%ID/g_{max} of ^{18}F-FMISO			
	Day 0	Day 3	Day 14	Day 21
Mice 1	1.4	2.2	2.3	3.5
Mice 2	1.5	2.1	2.3	6.3
Mice 3	1.5	2.6	3.7	5.6
Mice 4	1.6	2	2.3	6.3
Mice 5	2.6	4.3	2.4	3.6

Table B. Quantitative value of ^{18}F-FMISO %ID/g_{max} in fulvestrant group

Fulvestran Group	%ID/g_{max} of ^{18}F-FMISO			
	Day 0	Day 3	Day 14	Day 21
Mice 1	1.5	1.3	3.3	6.2
Mice 2	1.6	0.9	1.3	1.7
Mice 3	1.7	1.3	1.5	5.2
Mice 4	1.8	0.8	2.3	2.3
Mice 5	1.2	1.8	3.5	3.8

Table C. Quantitative value of ^{18}F-FMISO T/M in vehicle group

Vehicle Group	T/M of ^{18}F-FMISO			
	Day 0	Day 3	Day 14	Day 21
Mice 1	1.4	1.4	1.2	1.9
Mice 2	1.4	1.6	1.8	3.7
Mice 3	1.3	1.6	1.7	3.2
Mice 4	1.5	1.1	1.1	5.2
Mice 5	1.4	1.5	2.1	1.9

Table D. Quantitative value of ^{18}F-FMISO T/M in fulvestrant group

Fulvestran Group	T/M of ^{18}F-FMISO			
	Day 0	Day 3	Day 14	Day 21
Mice 1	1	1	3	3.8
Mice 2	1.2	1	1	1
Mice 3	1	1	1.1	2.7
Mice 4	1.8	1.6	1.9	1.2
Mice 5	2	1.2	1.7	2.1
