# Supplementary material for: Comparison of 18F-FES, 18F-FDG, and 18F-FMISO PET Imaging Probes for Early Prediction and Monitoring of Response to Endocrine Therapy in a Mouse Xenograft Model of ER-Positive Breast Cancer
Source: PLoS One. 2016 Jul 28;11(7):e0159916. doi: 10.1371/journal.pone.0159916 (PMC4965120; doi:10.1371/journal.pone.0159916)
Supplement: S1 Table — (PDF) [file pone.0159916.s005.pdf]

Table A. Correlation data of ERα score and ^{18}F-FES uptake value (%ID/g_{max}, T/M).

^{18}F-FES uptake		ERα score
%ID/g_{max}	T/M	
4.9	5.1	1
0.2	2.5	0
0.5	2.2	0
0.4	2.6	0
4.9	4.4	2
5	4.5	3
5	4.7	3
4	5	3
